# Effect of dietary supplementation of yeast culture *Saccharomyces cerevisiae* in lactating female goats

**DOI:** 10.3389/fvets.2024.1482800

**Published:** 2024-11-12

**Authors:** Li Zhang, Ge Qin, Jiaxue Guo, Mingding Zhang, Guangxin E, Yongfu Huang, Yanguo Han

**Affiliations:** Chongqing Key Laboratory of Forage and Herbivore, Chongqing Engineering Research Centre for Herbivores Resource Protection and Utilization, College of Animal Science and Technology, Southwest University, Chongqing, China

**Keywords:** yeast culture, lactation, weight, metabolism, female goats

## Abstract

This study was designed to investigate the effects of adding a novel yeast culture, *Saccharomyces cerevisiae* refermented sorghum distiller's dried grains with solubles (SSDDGS), to the diets of lactating female goats on lactation performance and lamb growth performance. We divided 10 lactating Dazu black goats of similar age, weight, and offspring into two groups: one fed a pelleted diet with 50 g/day SSDDGS (ET), and the other without SSDDGS as a control (EC) for 7 weeks. We monitor the weight changes of each goat and collect blood and milk samples from experimental ewes at specific times for hormone and milk composition determination. We use ultra-performance liquid chromatography tandem mass spectrometry (UPLC-MS/MS) to detect metabolites in the serum of lactating ewes. Our results showed that SSDDGS supplementation significantly reduced female goats' average daily weight loss during weeks 2–4 of lactation and increased serum IGF-1 and prolactin levels at week 4 (*p* < 0.05). SSDDGS supplementation in early lactation significantly increased milk protein, lactose, and ash content (*p* < 0.05). UPLC-MS/MS analysis showed that SSDDGS changed the levels of 58 metabolites in the serum of lactating goats. These metabolites were mainly involved in the sohingolipid signaling pathway, and cysteine, methionine, and sphingolipid metabolism. In summary, Yeast culture SSDDGS reduced weight loss, enhanced milk quality, and modified metabolic profiles in early lactation goats, providing insight into the potential regulatory role and mechanism of yeast culture in lactation female goats.

## 1 Introduction

The low fertility rate of goats poses a major challenge to the development of the goat industry. In addition to the number of lambs, the survival rate of lambs is also an extremely important reproductive trait, while the breast milk is the main source of nutrition for lambs. The digestive and circulatory system of female goats are weak in the early stages of lactation, and undergo significant metabolic shifts and energy allocation changes including weight loss, increased glucose intake, and compromised immunity (1). Insufficient or unbalanced intake of nutrients from feed will easily lead to a decline in the health status of female goats, which cannot meet the needs of rapid growth and development of lambs, causing slow growth and even death of lamb (2). Therefore, it is critical to design a feed suitable for lactating female goats to improve lactation performance in early lactation.

Yeast culture (YC) mainly consists of yeast extracellular metabolites, modified medium after fermentation and a small amount of inactive yeast cells. Supplementing YC in ruminant diet can improve the rumen pH environment, the efficiency of crude fiber digestibility and growth performance (3, 4). YC can increase body score, milk production and quality in the early lactation stage of dairy cows and sows (5–8). *Saccharomyces cerevisiae* refermented sorghum distiller's dried grains with solubles (SSDDGS) is a novel YC that is produced by re-fermentation of the distiller's grains substrate, which greatly increased the yield and reduced the cost of yeast culture production. The supplementation of SSDDGS in pig diet improved the lactation performance of sows (9). However, the study on the effect of the novel YC SSDDGS on the goat lactation performance remains uncertain.

Therefore, this study aims to determine the effect of supplementing a novel yeast culture SSDDGS in lactating goat diet on the weight loss of female goat, lactation performance, hormones and metabolites.

## 2 Materials and methods

### 2.1 Animal handling and sample collection

All animal tests and handling adhered to the regulations set forth by the Southwestern University Institutional Animal Care and Use Committee (IACUC-20210515-05). The feeding experiment was conducted at Tengda Animal Husbandry, Inc. in Chongqing, China. Ten lactating female goats who had just given birth with comparable weights were randomly divided them into two groups, and each female goats with twin lambs. One group served as the EC group and was fed a full-value pelleted diet (906) procured from Pizhou Xiaohe Technology Development Ltd, and the nutritional level of the diets are shown in Table 1. The other group constituted the ET group received a full-value pelleted diet (906) supplemented with 50 g/d novel yeast culture of *Saccharomyces cerevisiae* refermented sorghum distiller's dried grains with solubles (SSDDGS). Both groups of lactating female goats were given the same weight of feed twice daily at 7 a.m. and 3 p.m.

**Table 1:** Nutrient level of full-value pelleted diet fed to lactating female goats (dry matter basis).

**Nutrient level**	**Content**
Digestible energy, MJ/kg	11.1
Crude protein, %	12.5
Crude fiber, %	15.7
Crude ash, %	10.0
Ca, %	1.0
P, %	0.6

The experimental period started after 1 week of prefeeding, and the formal feeding period was 7 weeks, from weeks 2 to 8 of lactation. Peripheral blood and milk samples of lactating female goats were collected at week 4 and 8 of lactation. Blood samples were placed overnight at 4°C and then centrifuged at 3,000 rpm for 10 min, and the serum sample was collected. The weight of lactating female goats and offspring lambs was recorded at 4 and 8 weeks of lactation, and the ADL (average daily weight loss) of female goats and ADG of offspring lambs at 2–4 and 2–8 weeks of lactation.

### 2.2 Determination of hormones and milk composition

These concentrations of serum growth hormone releasing hormone (GHRH), somatostatin (SS), insulin-like growth factor-1 (IGF-1), prolactin (PRL), growth hormone (GH) in lactating female goats were quantified at week 4 and 8 of lactation via radioimmunoassay at the Sino-British Institute of Biotechnology in Beijing, China. Additionally, these milk components, including milk fat, protein, lactose, ash content and dry matter, were tested using an automatic milk composition analyzer (LACTOSCAN MCC 50, Milkotronic, Bulgaria).

### 2.3 Widely targeted metabolomics analysis of goat serum

Metabolites from the serum of lactating goat were determined by UPLC-MS/MS, according to the previous studies (10–12). Briefly, 50 μL of the sample and 300 μL of an extraction solution (comprising CAN and Methanol in a 1:4 ratio; V/V) containing internal standards was centrifuged at 12,000 rpm at 4°C and then the supernatant were used for UPLC-MS analysis. The samples were analyzed using a UPLC system that featured a UPLC column (2.1 mm × 100 mm, 1.8 μm; Waters ACQUITY UPLC HSS T3 C18). The column temperature was maintained at 40°C and the flow rate was set at 0.4 mL/min. The solvent system consisted of A (water with 0.1% formic acid) and B (acetonitrile with 0.1% formic acid). The gradient program of the system was as follows: 95:5 V/V at 0 min; 10:90 V/V at 11.0 min, 10:90 V/V at 12.0 min; 95:5 V/V at 12.1 min; and 95:5 V/V at 14.0 min. The injection volume was 2 μL. To ensure system stability, one QC sample was introduced every 10 test samples.

Analysis of the samples was conducted using an MS/MS system equipped with an ESI Turbo Ion-Spray interface, which operated in both positive and negative ion modes. The operational parameters of the ESI source were as follows: source temperature, 500°C; ion spray voltage (IS), 5,500 V (positive) and −4,500 V (negative); and ion source gases I, II, and curtain gas were maintained at 55, 60, and 25.0 psi, respectively.

### 2.4 Statistical analysis

ADL of female goat, ADG of lambs, hormone concentrations, milk composition data were analyzed using a general linear model in GraphPad Prism8.0.1 software (San Diego, CA, USA), with treatment and time as the fixed factors, and the individual samples as a random factor. The interaction of treatment and time was included in the model. These differences between the means were analyzed using Duncan's multiple comparisons.

Multivariate statistical analysis included unsupervised principal component analysis (PCA) and supervised orthogonal partial least squares-discriminant analysis (OPLS-DA). The variable importance in *p* ≤ 0.05, projection (VIP) ≥ 1 and log2FC (fold-change) ≤ 0.67 or log2FC ≥ 1.5 represented a significant difference in metabolites between the ET group and the EC group. The identified metabolites were annotated using the KEGG compound database, and KEGG pathways were used for metabolite set enrichment analysis. A *p*-value obtained from the hypergeometric test of < 0.05 indicated biological significance. *P* < 0.05 was considered to be statistically significant. Results are expressed as the mean ± the standard error (SEM).

## 3 Results

### 3.1 Changes in weight of lactating female goats and offspring lambs

The average daily weight loss (ADL) of lactating female goats in the experimental treatment (ET) group was significantly lower during week 2–4 of lactation than that in the experimental control (EC) group (Table 2, *p* < 0.05). There was no significant difference in the average daily weight gain (ADG) of offspring lambs between the ET and EC groups during week 2–4 or 2–8 of lactation (Table 2, *p* > 0.05).

**Table 2:** Effects of SSDDGS on the ADL of the lactation female goats and the ADG of offspring lambs.

**Item**	**Week 2–4 of lactation**		**Week 2–8 of lactation**		**SEM**	***p*** **-value**		
	**ET**	**EC**	**ET**	**EC**		**Treatment**	**Time**	**Treatment × Time**
ADL of female goats, kg	0.08^a^	0.22^b^	0.08^a^	0.10^ab^	0.02	0.016	0.048	0.070
ADG of lambs, kg	0.10^a^	0.08^a^	0.09^a^	0.10^a^	0.01^a^	0.447	0.732	0.035

^a, b^Different superscript letters represent significant differences in the same row (p < 0.05). SSDDGS, Saccharomyces cerevisiae refermented sorghum distiller's dried grains with solubles; ADG, Average daily weight gain; ADL, Average daily weight loss; ET, SSDDGS treatment group; EC, control group.

### 3.2 Changes in serum hormones in lactating female goats

Serum insulin-like growth factor-1 (IGF-1) or prolactin (PRL) concentration in the ET group was significantly higher than that in the EC group at week 4 of lactation (Table 3, *p* < 0.05). In addition, in the ET group, IGF-1 concentration at week 8 of lactation was significantly lower than that at week 4 of lactation (Table 3, *p* < 0.05).

**Table 3:** Effects of SSDDGS on the hormone concentration of female goats.

**Hormone concentration**	**Week 4 of lactation**		**Week 8 of lactation**		**SEM**	***p*** **-value**		
	**ET**	**EC**	**ET**	**EC**		**Treatment**	**Time**	**Treatment × time**
GHRH, ng/mL	42.90	40.70	47.58	42.29	1.18	0.114	0.180	0.500
SS, pg/mL	20.78	21.96	21.63	21.83	0.47	0.500	0.723	0.633
IGF-1, ng/mL	185.78^a^	154.67^b^	156.89^b^	144.10^b^	4.56	0.003	0.006	0.161
PRL, μIU/mL	245.97^a^	223.17^b^	255.53^ab^	240.10^ab^	3.92	0.007	0.048	0.561
GH, ng/ml	4.65	4.62	5.58	4.72	0.23	0.353	0.283	0.382

^a, b^Different superscript letters represent significant differences in the same row (p < 0.05). GHRH, growth hormone releasing hormone; SS, somatostatin; IGF-1: insulin-like growth factor-1; PRL, prolactin; GH, growth hormone.

### 3.3 Effects of SSDDGS on milk quality in lactation female goats

Milk composition was more abundant in the ET group compared to the EC group at week 4 of lactation. The content of milk protein, milk lactose or ash in the ET group was significantly higher than that in EC group at week 4 of lactation (Table 4, *p* < 0.05).

**Table 4:** Effects of SSDDGS on the milk composition in lactating female goats.

**Milk composition**	**Week 4 of lactation**		**Week 8 of lactation**		**SEM**	***p*** **-value**		
	**ET**	**EC**	**ET**	**EC**		**Treatment**	**Time**	**Treatment × time**
Fat, %	4.92	4.80	6.35	5.88	0.32	0.648	0.061	0.786
Protein, %	3.60^a^	3.34^b^	3.50^ab^	3.30^ab^	0.05	0.026	0.477	0.768
Lactose, %	5.43^a^	5.04^b^	5.30^ab^	5.00^ab^	0.07	0.020	0.531	0.762
Ash content, %	0.33^a^	0.30^b^	0.32^ab^	0.30^ab^	0.00	0.018	0.539	0.712
Dry matter, %	14.29	13.51	15.02	14.97	0.28	0.443	0.054	0.494

^a, b^Different superscript letters represent significant differences in the same row (p < 0.05).

### 3.4 Effects of SSDDGS on metabolites of goats

**Figure 1:**
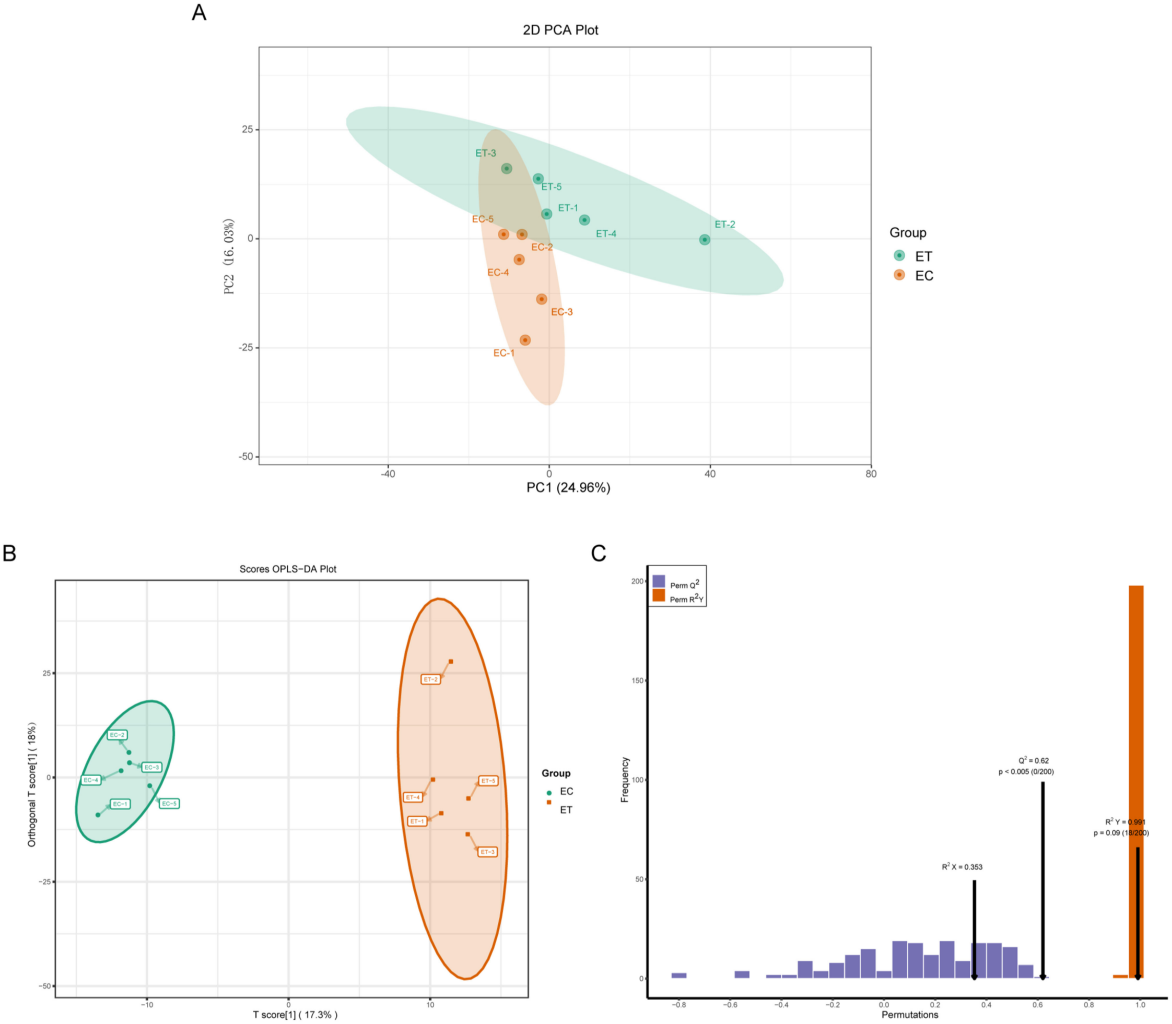
Effects of SSDDGS on goat serum metabolites were analyzed by principal component analysis (PCA) and orthogonal partial least squares-discriminant analysis (OPLS-DA). **(A)** PCA diagram of ET and EC groups. PC1 is the first principal component, and PC2 is the second principal component. each dot represents a sample, and the same color represents the same group; **(B)** OPLS-DA score plot of ET and EC groups, and the percentage represents the interpretation rate of the component to the data set; **(C)** OPLS-DA verification diagram, R^2^X, R^2^Y and Q^2^ are the prediction parameters for evaluating the OPLS-DA model, and when 1 > R^2^ Y and Q^2^ > 0.4, the models were determined to be stable and reliable.

Effects of SSDDGS on metabolites of goats was analyzed by ultra-performance liquid chromatography tandem mass spectrometry (UPLC-MS/MS). The principal component analysis (PCA) score plot (Figure 1A) revealed that the model interpretation rates for PC1 and PC2 were 24.96% and 16.03%, respectively, with a clear separation of samples between the two groups. The OPLS-DA results mirrored those obtained from PCA, with a principal component of prediction in the ET and EC groups accounting for 17.3% of the model interpretation rate (Figure 1B). Furthermore, the OPLS-DA model displayed the predictive parameters of Q^2^ = 0.62 and R^2^Y = 0.991, indicating the stability and reliability of the model (Figure 1C).

**Figure 2:**
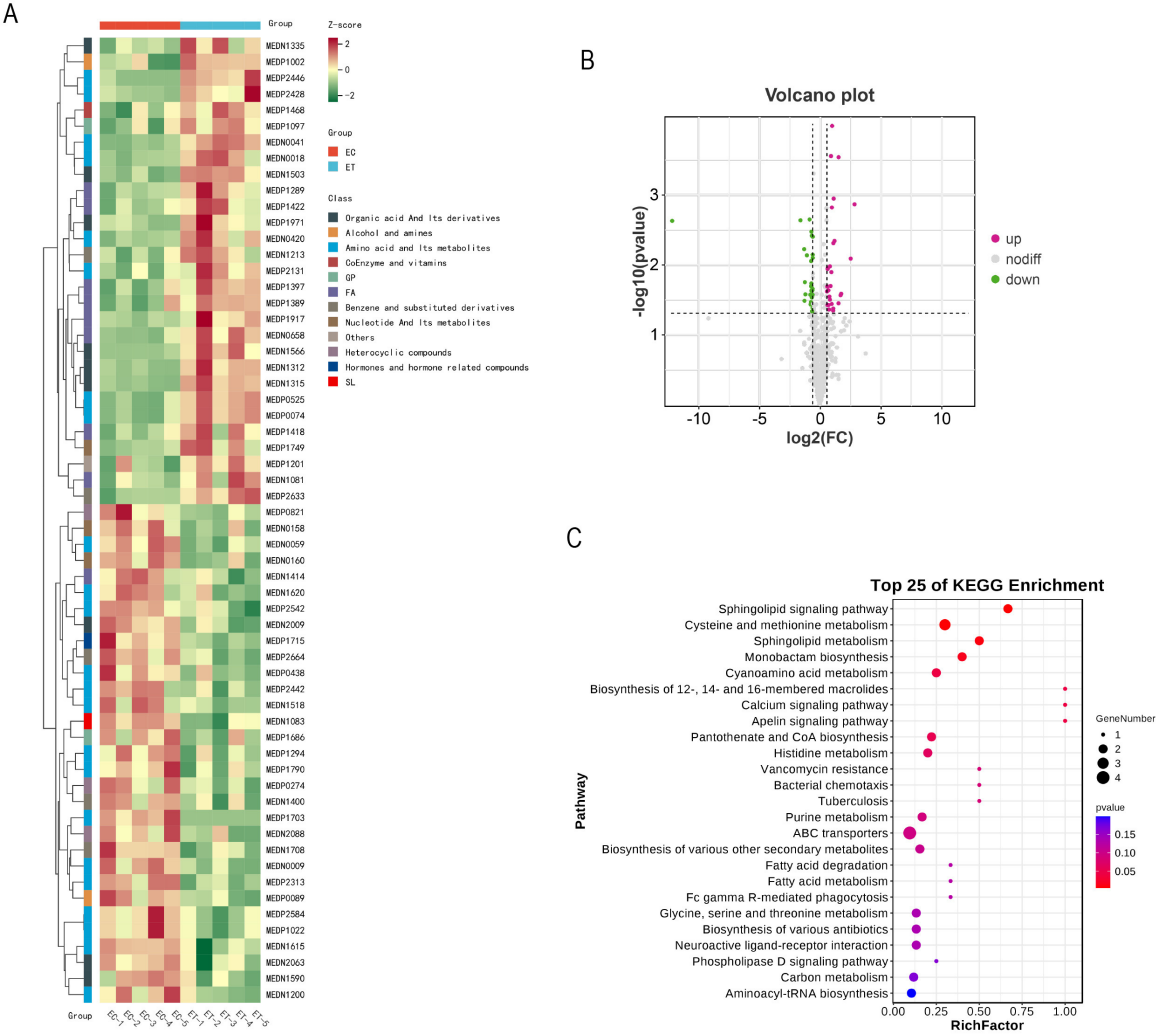
Effects of SSDDGS on metabolites of goats. **(A)** Differential metabolite clustering heat map. **(B)** Volcanic diagram of differential metabolites. **(C)** KEGG enrichment map of differential metabolites. The color of the dot represents the size of *P*-value, and the deeper the red color on behalf of the enrichment of the more significant.

The identification of 60 significantly different metabolites in the ET group compared with the EC group (Table 5, Figure 2A). Among these, 31 metabolites were downregulated, whereas 29 were upregulated (Figure 2B). The various types of metabolites include L-leucylglycine, aspartic acid, sphingosine 1-phosphate, cinnamyl glycine, serine and hexanoyl glycine.

**Table 5:** Different metabolites of ET group compared with EC group.

**Compounds**	**Class**	**VIP**	***P*-value**	**log1.5FC**	**Type**
1,6-Di-O-phosphono-D-fructose	Organic acid and its derivatives	1.44	0.05	−20.76	Down
12,13-DiHOME	FA	1.49	0.05	−2.72	Down
12-HHT	FA	1.55	0.05	−2.19	Down
2-(4-hydroxyphenyl) propionate	Benzene and substituted derivatives	1.59	0.04	−2.16	Down
2-Butyl-3-(4-hydroxybenzoyl)benzofuran	Benzene and substituted derivatives	1.60	0.04	−2.10	Down
2-Hydroxy-3-Methyl Butanoic Acid	Organic acid and its derivatives	1.60	0.04	−2.04	Down
2-Hydroxymelatonin	Hormones and hormone related compounds	1.60	0.04	−1.82	Down
2-hydroxyphenylacetic acid	Organic acid and its derivatives	1.60	0.04	−1.45	Down
3-(4-Hydroxyphenyl)-1-propanol	Organic acid and its derivatives	1.61	0.04	−1.34	Down
3-Methylcrotonyl Glycine	Organic acid and its derivatives	1.61	0.04	−1.31	Down
4-Hydroxy-3-methylbenzoic acid	Organic acid and its derivatives	1.62	0.04	−1.25	Down
4-Hydroxyquinoline	Benzene and substituted derivatives	1.62	0.03	−1.23	Down
4-Methoxysalicylic Acid	Benzene and substituted derivatives	1.68	0.03	−1.22	Down
6-Methylnicotinamide	Heterocyclic compounds	1.69	0.03	−1.18	Down
8,8a-deoxy-oleane	Others	1.71	0.03	−1.18	Down
Acetylcholine	Alcohol and amines	1.72	0.03	−1.18	Down
Acetylvaline	Amino acid and its metabolites	1.72	0.03	−1.17	Down
Carnitine C11:1	FA	1.73	0.03	−1.17	Down
Carnitine C16:0	FA	1.73	0.03	−1.12	Down
Carnitine C18:0	FA	1.74	0.03	−1.11	Down
Carnitine C18:1:2DC	FA	1.74	0.03	−1.08	Down
Carnitine C6:0	FA	1.75	0.03	−1.07	Down
Carnitine C8-OH	FA	1.75	0.02	−1.05	Down
Cyclo(Ala-Pro)	Amino acid and its metabolites	1.76	0.02	−1.03	Down
Cytidine 5′-diphosphate	Nucleotide and its metabolites	1.78	0.02	−1.03	Down
Deoxyguanosine	Nucleotide and its metabolites	1.78	0.02	−1.02	Down
Gln-Gly	Amino acid and its metabolites	1.79	0.02	−1.01	Down
Glu-Val	Amino acid and its metabolites	1.81	0.02	−1.00	Down
Gly-Gln	Amino acid and its metabolites	1.82	0.02	−1.00	Down
Guanosine	Nucleotide and its metabolites	1.87	0.02	1.02	Up
Hexadecanedioic acid	FA	1.90	0.02	1.06	Up
Hexanoyl Glycine	Amino acid and its metabolites	1.90	0.02	1.09	Up
Ile-Pro-Ile	Amino acid and its metabolites	1.90	0.02	1.11	Up
Imidazole-4-methanol	Heterocyclic compounds	1.90	0.01	1.12	Up
Indole-2-Carboxylic Acid	Heterocyclic compounds	1.91	0.01	1.20	Up
L-Aspartic Acid	Amino acid and its metabolites	1.92	0.01	1.23	Up
L-Isserine	Amino acid and its metabolites	1.94	0.01	1.30	Up
L-Serine	Amino acid and its metabolites	1.96	0.01	1.35	Up
L-tyrosine methyl ester 4-sulfate	Amino acid and its metabolites	1.97	0.01	1.41	Up
LPE(15:0/0:0)	GP	2.00	0.01	1.42	Up
Leu-Gly	Amino acid and its metabolites	2.01	0.01	1.51	Up
Leu-Met	Amino acid and its metabolites	2.01	0.01	1.57	Up
MG(18:2/0:0/0:0)	GL	2.02	0.01	1.57	Up
Methyldopa	Amino acid and its metabolites	2.04	0.00	1.63	Up
N-Acetyl-L-alanine	Amino acid and its metabolites	2.05	0.00	1.68	Up
N-Acetylglycine	Amino acid and its metabolites	2.10	0.00	1.70	Up
N-Acetylhistamine	Alcohol and amines	2.11	0.00	1.73	Up
N-Cinnamylglycine	Organic acid and its derivatives	2.15	0.00	1.87	Up
N-Formylmethionine	Amino acid and its metabolites	2.17	0.00	1.88	Up
N-Propionylglycine	Amino acid and its metabolites	2.20	0.00	1.90	Up
Octadecanedioic acid	Organic acid and its derivatives	2.20	0.00	1.94	Up
Pantothenol	CoEnzyme and vitamins	2.21	0.00	2.07	Up
Phe-Thr	Amino acid and its metabolites	2.22	0.00	2.63	Up
Salicyluric acid	Benzene and substituted derivatives	2.22	0.00	2.64	Up
Sphingosine 1-phosphate	SL	2.27	0.00	2.91	Up
Thr-Phe	Amino acid and its metabolites	2.27	0.00	3.00	Up
Tricarballylic acid	Organic acid and its derivatives	2.33	0.00	4.35	Up
Cyclo(pro-pro)	Amino acid and its metabolites	2.39	0.00	4.89	Up

The metabolic pathways of these 60 different metabolites were analyzed by KEGG enrichment analyses. The results revealed the involvement of 25 metabolic pathways, among which the with the *p*-value closest to 0 and the highest number of differentially significant metabolites included sohingolipid sianaling pathway, cysteine and methionine metabolism and sphingolipid metabolism (Figure 2C).

**Figure 3:**
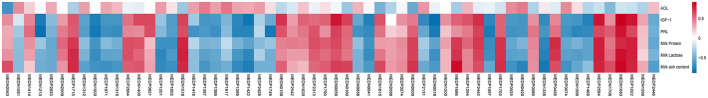
Pearson correlation analysis between phenotypic data and serum differential metabolites in female goats. Phenotypic data were obtained from the 4th week of lactation, Pearson correlation coefficient >0.7 with a *P* < 0.05 were considered significant.

Meanwhile, to further analyzed the potential correlations between female goat phenotypic data and differential metabolites in serum, we performed Pearson correlation analysis (Figure 3). L-leucylglycine, aspartic acid, sphingosine and 1-phosphate exhibited positive correlations with serum biochemical index (IGF-1, PRL) and milk composition (protein, lactose, and ash content) of female goats (*P* < 0.05). In addition, monoglyceride, monoglyceride and amino acid (AA) such as cinnamyl glycine, serine and hexanoyl glycine had negative significant correlations with serum biochemical index (IGF-1, PRL) and milk composition (protein, lactose, and ash content) of female goats (*P* < 0.05).

## 4 Discussion

Yeast culture contains a large number of beneficial microorganisms and easily digestible small molecules, which is very beneficial to improve animal health and production performance. During early lactation, female goats experience increased energy expenditure, heightened production of volatile fatty acids, and decreased body weight (1). Supplementation of YC in diet can enhance nutrient digestibility and promoting animal growth by increasing the diversity and abundance of rumen microorganisms (13–15).

In this study, the supplementation of SSDDGS in diet significantly reduced the ADL of lactating female goats during early lactation. Similarly, Song et al. reported that incorporating YC into the diet improved the rumen microbial community and increased the ADG of lambs (16). Additionally, in this study, the supplementation of SSDDGS in diet significantly increased the serum IGF-1 concentration during early lactation. IGF-1 is an endocrine hormone primarily produced by the liver and promotes animal growth and development, which is involved in lipid metabolism, insulin secretion, and glucose uptake (17). These results indicate that the dietary supplementation of SSDDGS reduced weight loss of lactating female goats during early lactation through the role of IGF-1.

Supplementation of YC in diet can effectively improve animal lactation performance. In this study, the supplementation of SSDDGS in diet significantly increased the milk protein, milk lactose and ash content during early lactation, although SSDDGS supplementation in diet did not increase ADG of offspring lambs in lactation, which was an important indicator of milk production in female goats. These studies are consistent with previous studies. YC supplementation increased milk protein content and reduced the incidence of mastitis in cows (18). Similarly, the milk protein and fat content in cows increased after YC supplementation, particularly during early lactation, without affecting overall milk production (19). Supplementation of YC or *Saccharomyces cerevisiae* in diet increased milk yield and fat production in lactating female goats (20, 21). Additionally, in this study, the supplementation of SSDDGS in diet significantly increased the serum PRL levels during early lactation. PRL is vital for sustaining mammary gland function, milk yield and milk quality in ruminants. Chen et al. found that PRL increased milk protein synthesis by stimulating mammary epithelial cell metabolism (22). These results suggest that supplementation of YC in diet improved the milk quality of lactating female goats during early lactation through the role of PRL.

The supplementation of SSDDGS in diet played a crucial role in the inflammation and the immune system of organisms based on metabolome analysis. Supplementing with *Saccharomyces cerevisiae* fermentation postbiotics in calves increased resistance to bovine respiratory disease through the systemic and mucosal immune responses (23). *Saccharomyces cerevisiae* culture can reduce the number of somatic cells in milk and enhance the antioxidant capacity in cow under heat stress (24). During lactation, there is a high demand for glucose for milk synthesis and fatty acid production. However, excessive energy consumption can lead to mastitis, and excess production of free fatty acids results in the formation of ketone bodies, which can reduce the immunity of female goats (25). From the results of this study, we observed a significant upregulation of Carnitine C11:1 and Carnitine C18:1:2DCs in the SSDDGS group, which are involved in fatty acid metabolism and degradation pathways. The expression abundance of Sphingosine 1-phosphate was significantly downregulated and involved in the Fc gamma R-mediated phagocytosis and tuberculosis pathway. Previous reports have shown that carnitine supplements increase the flux of metabolites through pyruvate carboxylase, thereby increasing insulin secretion and liver glucose output(26). In the mitochondria isolated from pig liver fed carnitine, it was found that the amount of mitochondrial pyruvate carboxylase increased threefold. Pigs fed carnitine are better able to utilize fat to obtain energy, transfer carbon to amino acid synthesis, and save branched chain amino acids used for protein synthesis (27). Similarly, another study found that high levels of carnitine can enhance lipid metabolism in sheep, thereby altering lactation performance (28). However, there has been no research on supplementing carnitine in the diet of goats, and these reports suggest that carnitine may be the main substance that improves goat lactation performance.

In addition, deoxyguanosine, Gly Leu, Phe Thr, and sphingosine 1-phosphate were significantly positively correlated with IGF-1 and PRL levels. Related to this, Purine metabolism, ABC transporters, Calcium signaling pathway and Apelin signaling pathway were significantly enriched. Luo et al. investigated the effects of fermented soybean meal (FSBM) rich in isoflavone glycosidic ligands at different levels on ewes from late pregnancy to early lactation. Feeding ewes with FSBM6 reduced the concentrations of hydrogen peroxide and deoxyguanosine in the placenta, improved the antioxidant capacity of both the mother and placenta, and improved serum hormones and milk quality (29). Sphingosine kinase (SK) catalyzes the formation of sphingosine-1-phosphate (S1P), which plays an essential role in cell growth and survival (30). Döll et al. found that prolactin (PRL) activates SK-1 but not SK-2 isoforms in a human breast cancer cell line (MCF7). The delayed activation of SK-1 results from up-regulation of mRNA and protein expression and is due to increased activity of the SK-1 promoter by a mechanism involving STAT5 activation as well as protein kinase C and classical mitogen-activated protein kinases. The delayed activation of SK-1 results from up-regulation of mRNA and protein expression due to increased SK-1 promoter activity and involves STAT5 activation as well as protein kinase C and classical mitogen-activated protein kinase (31). This suggests that sphingosine-1-phosphate is likely to play an important role in lactation performance.

## 5 Conclusion

This study demonstrates that the dietary supplementation of yeast culture SSDDGS reduced weight loss and improved milk quality of female goats in the early stage of lactation. Furthermore, SSDDGS may play a vital role in energy metabolism and immune responses primarily through modulation of bile acid and caffeine metabolic pathways. This study will help us better understand the effects and mechanism a novel yeast culture SSDDGS in lactating female goats.

## Data Availability

The datasets presented in this study can be found in online repositories. The names of the repository/repositories and accession number(s) can be found in the article/supplementary material.
